# The Dilemma in the Diagnosis of Paratesticular Lesions

**DOI:** 10.7759/cureus.22783

**Published:** 2022-03-02

**Authors:** Sajika P Dighe, Raju K Shinde, Sangita J Shinde, Pratikshit S Raghuwanshi

**Affiliations:** 1 General Surgery, Datta Meghe Institute of Medical Sciences University (DMIMSU), Wardha, IND; 2 Pharmacology and Therapeutics, Datta Meghe Institute of Medical Sciences University (DMIMSU), Wardha, IND

**Keywords:** benign tumour, epididymis, extra-testicular tumor, paratesticular tumor, adenomatoid tumour

## Abstract

Intra-scrotal swellings are commonly encountered all over the globe. Common causes include hydroceles, hernias, lipoma, cysts, etc. Rarely manifested as scrotal swelling is paratesticular swelling arising outside the testis. Clinically, they may mimic a tumour arising from testis either benign or malignant with grave consequences. Diagnosis hence is of paramount importance. It has to be ruled out as a diagnosis of exclusion. Knowledge of such swelling those other than the ones originating from the testis is thus crucial in order to have a better patient outcome in the absence of any mandated published guideline.

## Introduction

Infrequent in incidence, para/extra-testicular neoplasms are clinically noteworthy affecting all males. These tumours are asymptomatic, benign with/without harmful consequences manifesting as a small swelling in the inguinoscrotal region [1]. The global incidence is less than 5% of all intra-scrotal lesions, out of which 80% are benign and 20% are malignant [2]. Para-testicular area includes spermatic cord, epididymis, vestigial remnants and tunica vaginalis in males. In females, these tumours are lesser known as para-ovarian tumours in areas adjacent to the uterus, fallopian tubes and ovaries [1,2]. Maximum male patients encountered lesions in the groin area mostly with abnormalities of the epididymis (spermatoceles, cysts), fluid accumulation in tunica vaginalis (hydrocele), hernias (inguinal/scrotal) and inflammatory conditions (epididymitis, orchitis). Prevalence of primary malignancy of the paratesticular region is sparse, ranging from 3% to 6% [1]. Among all, the most frequent is lipoma and commonly of spermatic cord. In the case of the epididymis, the most common benign tumour found is adenomatoid tumour and leiomyoma followed by rare being malignant sarcomas (liposarcoma, rhabdosarcoma and leiomyosarcoma) [1,3].

## Case presentation

A 40-year-old male labourer had painless, insidious, slow-growing swelling in the left scrotum for one year. Significant complaints of mild pain in the left scrotum while walking led to the detection of this swelling. Physical examination revealed non-tender, firm swelling felt just above the upper pole of the testis along the spermatic cord.

**Figure 1:**
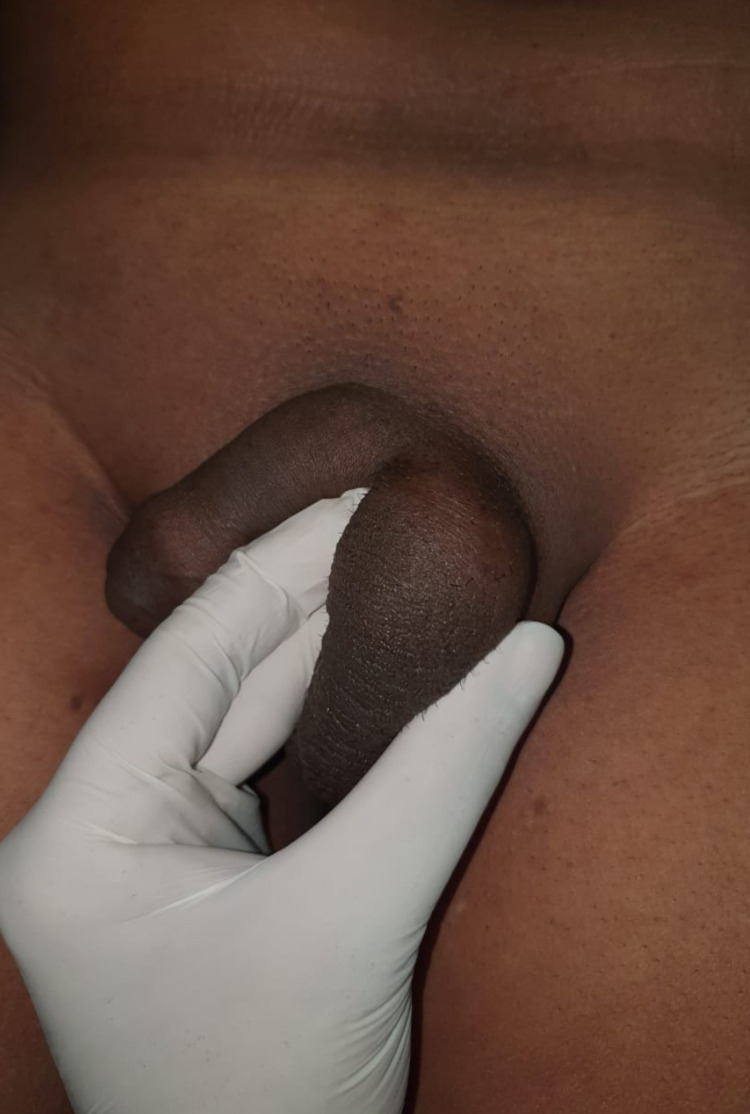
Clinical photograph of the left hemiscrotum with a visibly evident swelling.

USG scrotum unveiled isoechoic, homogeneous, avascular soft-tissue mass of 3.2cm x 3cm in left hemiscrotum, along spermatic-cord discrete from left testis suggestive of a probable benign epididymal adenomatoid tumour (Figure 1). As the benign nature of swelling was established on ultrasound, specific tumour markers of testicular malignancy were not sought and exploration of the left hemiscrotum under spinal anaesthesia was performed.

**Figure 2:**
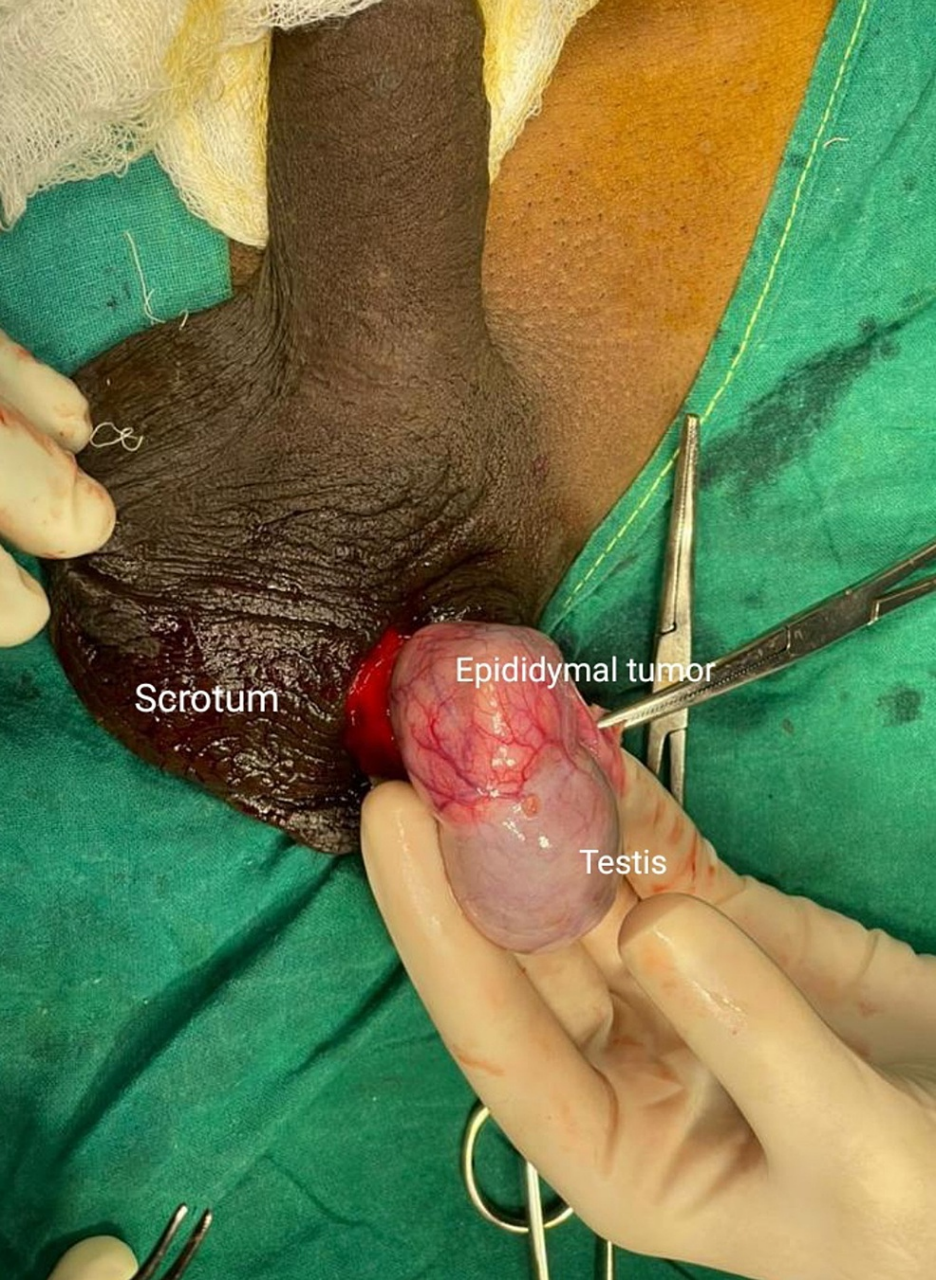
Intra-operative identification of the tumour lying at the upper pole of the left testis.

Intra-operatively, 3cm oval mass was discovered at the upper pole of the testis arising from the head of the epididymis although lying separately from the testis. Hence an en bloc excision of the mass was accomplished without damaging structures followed by a frozen section which confirmed the diagnosis of an adenomatoid tumour. The macroscopic cut section revealed a solid, homogenous, smooth-surfaced, whitish-yellowish lesion. Histopathology confirmed the diagnosis of adenomatoid tumour of epididymis with the presence of tubular epithelium and stromal cuboidal cells. Immunohistochemical positivity for adenomatoid-specific markers (Calretinin) was further added to the confirmation of diagnosis (Figure 2). The patient had an uneventful post-operative period.

## Discussion

Globally paratesticular tumours are rarely encountered with an overall incidence of less than 5%. Data regarding the incidence of paratesticular tumours in Toto or epididymal tumours per se in India are also missing due to a lack of conducted studies and knowledge about these cases. Lipoma of the spermatic cord is a popular benign paratesticular tumour and in the epididymis, the most common is adenomatoid tumour followed by leiomyoma, fibroma, haemangioma, neurofibroma and papillary cystadenoma. Such benign tumours also mimic an extra-testis (polyorchidism) [1,2]. In 2015, Khandeparker et al. conducted a six-year retrospective study in India in a tertiary care set-up that analysed the histopathological spectrum of tumours and tumour-like lesions of paratestis in 49,934 specimens. Results of distribution of paratesticular tumours (0.1%) in India were consistent with the available data of lipoma of paratestis being the most commonly encountered (6.4%) followed by adenomatoid tumour (2.1%) and leiomyoma (2.1%) of the epididymis [3,4]. A similar study was conducted in 2021 in North India showed consistent findings with Khandeparker et al. [5].

The adenomatoid tumour is commonly found benign tumour of epididymis with an incidence of 73% followed by papillary cystadenoma (11%), leiomyoma (9%) [6]. Embryology of adenomatoid was previously a subject of argument due to the hypothesis of its origin from either vascular endothelium, mesonephros or Mullerian epithelium. But the recent most documents have now affirmed its mesothelial origin. A hormone-independent tumour, its typical location is in the epididymis, although can rarely arise in tunica, testis, spermatic cord, prostate and ejaculatory ducts. Few inconsistent reports have shown adenomatoid involving adrenals, lymph nodes, pancreas, pleura and mediastinum. Inside epididymis, emergence from the tail/body is four times more often than its head. In our case, the tumour emerged from the head. Commonly it is a solitary tumour with an unknown preponderance in the left hemiscrotum [1,2,6,7]. It was discovered by Kontos et al. in 1945 and was described then as a tiny, firm, symptomless lesion inside the scrotum, common in males of the 30-50 years age group [8].

These tumours are not age influenced but are found in ages between 18 and 80 years [1,2,6,7]. Patoulias et al. have reported such cases in patients as young as 16 years and 12 years respectively [7]. These tumours are incidental, asymptomatic or patients might just complain of minimal pain during exercising or are noticed after an unrelated acute traumatic or inflammatory episode, either oval or round if emerging from epididymis or testis, respectively. Their size is small with an average diameter between 0.2 and 5 cm. Few inconsistent reports have suggested tumours up to 12 cm [1,2,7]. Clinical differentials include all practical scrotal swellings including lipoma, cyst, fibroma, sarcoma, granuloma, hematoma and metastasis. In middle-aged patients, it is important to rule out evidence of seminoma [4,7].

Ultrasonography is the initial investigation of choice as it is easily available, cost-effective, and diagnostic with outrageous sensitivity and specificity. It differentiates between a benign or malignant lesion, demarcates boundaries and describes echogenicity, vascularity, invasive behaviour and adjacent structures. If USG reveals a well bordered, solitary, homogenous, non-invasive lesion further use of CECT or MRI can be limited. If USG findings are unclear or doubtful, further radiology can be performed using CT or MRI and sending tumour markers for testicular malignancy for evaluation. FNAC can be performed but in over case was avoided since USG diagnosed a benign lesion which was then planned for excision [1,2,4,7].

Gross examination reveals a solitary, smooth, extra-testicular white-yellow nodule-like lesion. Microscopy reveals patterns of tubules, cords and nests with cuboidal epithelium and vacuolated/eosinophilic cytoplasm along with fibrous hyalinised stroma. Pathological differentials incorporate metastatic carcinoma, malignant mesothelioma, histiocytoid haemangioma and carcinoma of the rete testis. Here comes the use of Immunohistochemical analysis which helps in the final confirmation of diagnosis by differentiating benign from malignant and mesothelial from non-mesothelial. The mesothelial origin of adenomatoid is confirmed by the existence of Calretinin which is a calcium-binding protein of the S-100 family. It shows an exorbitant amount of sensitivity for the detection of mesothelial cells. Other marker includes HMB1, CK 5/6, vimentin, CK EMA and WT1 [4,7].

Treatment of choice is surgical which includes confirmative diagnosis by frozen section followed by complete resection of the tumour by scrotal approach without man-handling of adjacent structures. If the frozen section diagnoses a benign tumour, no further exploration is indicated as there is no evidence of recurrence reported in the literature of adenomatoid tumour to date [1-3,7].

## Conclusions

After acknowledging current literature we found sparse case reports of epididymal adenomatoid tumour in the last five years, specifically from India. Considering the rarity and importance of diagnosis to rule out malignancy, we are presenting this case with a motif of creating awareness regarding such benign tumours, which should be addressed during primary surveillance. The creation of appropriate guidelines for pre-operative, intra-operative and post-operative management of such cases is a must for correct diagnosis and overall prognosis of the patient.
